# Forward-viewing endoscopic ultrasound-guided biliary drainage for coil-induced choledochojejunal anastomosis stenosis

**DOI:** 10.1055/a-2598-4784

**Published:** 2025-06-03

**Authors:** Yasuhiro Komori, Susumu Hijioka, Shin Yagi, Mark Chatto, Takuji Okusaka, Yutaka Saito

**Affiliations:** 1 13874 Department of Hepatobiliary and Pancreatic Oncology, National Cancer Center Japan, Tsukiji, Chuo-ku,Tokyo, Japan; 2 37571 Department of Medicine, Makati Medical Center, Manila, Philippines; 3 13874 Endoscopy Division, National Cancer Center Japan, Tsukiji, Chuo-ku, Tokyo, Japan

A few reports have described bile duct obstruction caused by coil embolization for pseudoaneurysms 1 2 3 . The utility of rescue methods using forward-viewing endoscopic ultrasound (FVEUS) for choledochojejunal anastomosis stenosis (CJS) has recently been reported 4 5 . Herein, we report a case in which FVEUS-guided biliary drainage (FVEUS-BD) was successfully used to treat CJS caused by a coil ( Video 1 ).

Forward-viewing endoscopic ultrasound-guided biliary drainage resolved coil-induced choledochojejunal anastomosis stenosis in a patient with Roux-en-Y reconstruction.Video 1

A 73-year-old man with a history of distal gastrectomy and Roux-en-Y (RY) reconstruction for gastric cancer underwent pancreaticoduodenectomy for pancreatic head cancer complicated by a right hepatic artery pseudoaneurysm on postoperative day 12 with coil embolization. Two years postoperatively, the patient developed jaundice (total bilirubin: 4.8 mg/dL); computed tomography (CT) revealed CJS due to coil migration ( Fig. 1 ). A double-balloon endoscope (DBE) was used to approach the CJS. However, despite anastomosis visualization, the coil was not observed. The guidewire (GW) failed to pass through the coil, making biliary drainage unsuccessful. Thus, EUS-guided hepaticogastrostomy (EUS-HGS) was performed. A cholangioscope was advanced through the HGS fistula to attempt coil penetration with a GW but was unsuccessful. Although jaundice resolved with HGS, FVEUS-BD was attempted to achieve a stent-free state.

**Fig. 1:**
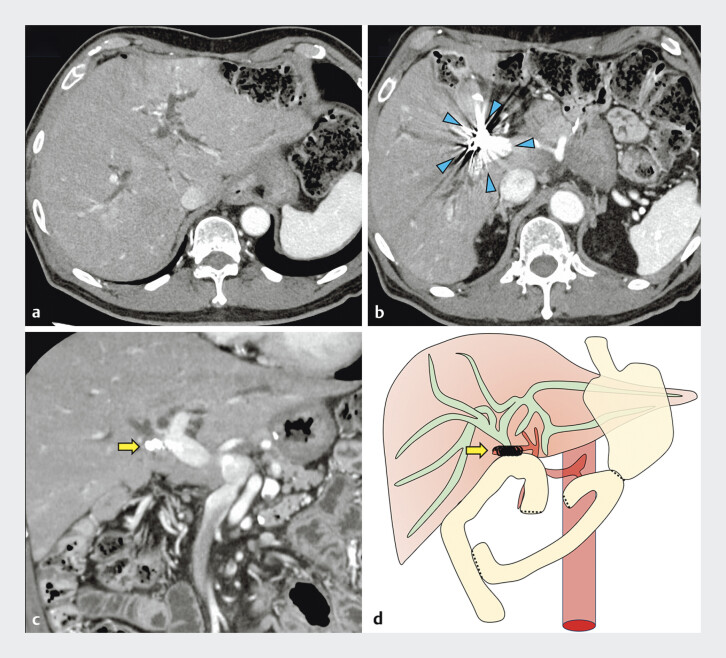
Choledochojejunal anastomotic stricture caused by coil embolization of the right hepatic artery. **a** Dilation of the intrahepatic bile duct. **b** Coil embolization of the right hepatic artery (arrowhead). **c** and **d** The choledochojejunal anastomosis is obstructed by the coil (arrow).

Considering the difficulty of advancing FVEUS (TGF-UC260J; Olympus) to the anastomosis because of RY reconstruction, DBE was initially used to reach the choledochojejunal anastomosis, and a GW was placed. FVEUS was then advanced along the GW to the anastomosis, revealing an obstructed bile duct beneath the coil that was punctured through the coil ( Fig. 2 ). After CJS dilation, one fully covered metal stent and one plastic stent were deployed ( Fig. 3 ). Six months later, both stents were removed and a stent-free state was achieved ( Fig. 4 ). This is the first report to describe using FVEUS-BD as a rescue technique for coil-induced CJS in a patient with RY reconstruction 4 .

**Fig. 2:**
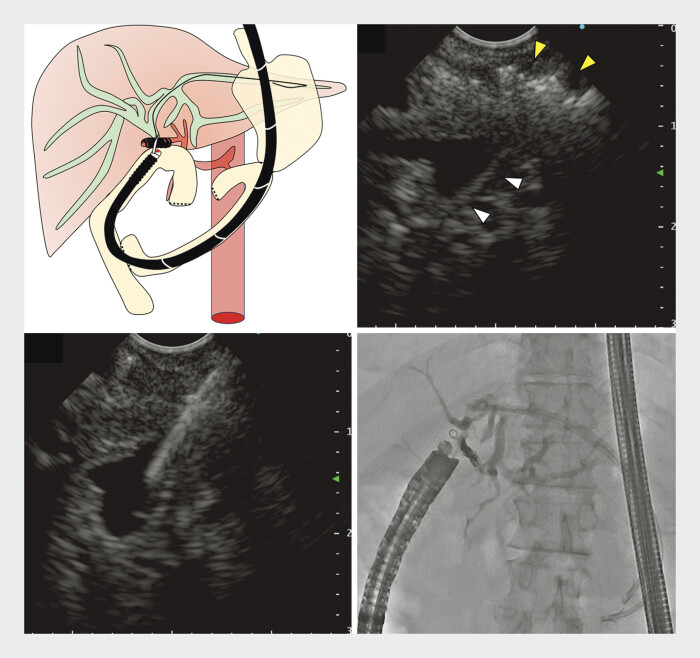
The procedure of drainage using FVEUS for coil obstruction of the choledochojejunal anastomosis. **a** Schematic of biliary drainage using FVEUS. **b** The FVEUS image shows the coil (yellow arrowhead) and the dilated bile duct (white arrowhead). **c** Puncture of the bile duct through the coil. **d** Post-puncture cholangiographic image. Abbreviation: FVEUS, forward-viewing endoscopic ultrasound.

**Fig. 3:**
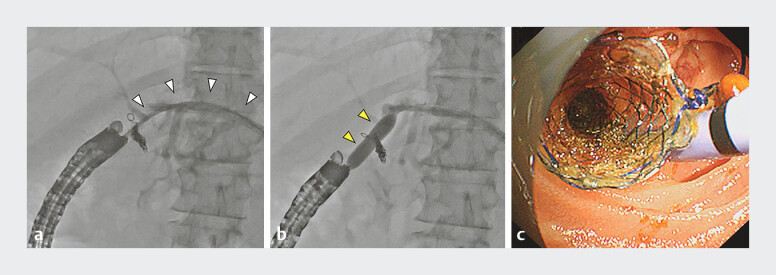
Dilating the anastomotic site and stent placement. **a** Dilation of the choledochojejunal anastomosis using a drill dilator (white arrowhead; Tornus ES, Asahi Intecc). **b** Dilation of the choledochojejunal anastomosis using a balloon dilator (yellow arrowhead; REN, Kaneka Medix Corporation). ( **c** ) Placement of a fully covered metal stent and a plastic stent.

**Fig. 4:**
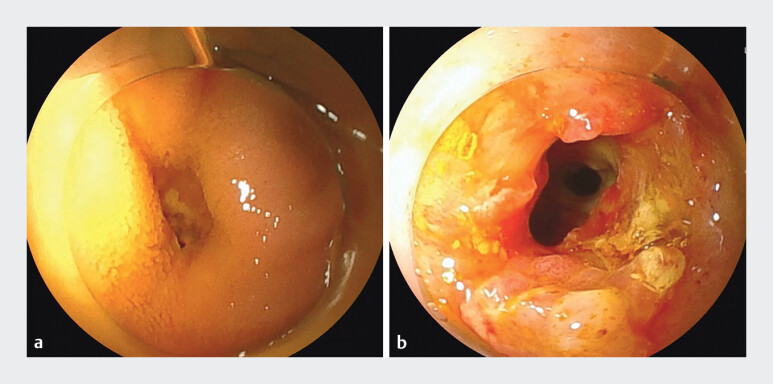
Images of the anastomotic site before and after the procedure. **a** Preprocedural image of the choledochojejunal anastomosis. **b** Post-FVEUS-BD image of the choledochojejunal anastomosis. Abbreviation: FVEUS-BD, forward-viewing endoscopic ultrasound-guided biliary drainage.

Endoscopy_UCTN_Code_TTT_1AR_2AK

## Notes

Endoscopy E-Videos https://eref.thieme.de/e-videos
*E-Videos* is an open access online section of the journal *Endoscopy* , reporting on interesting cases and new techniques in gastroenterological endoscopy. All papers include a high-quality video and are published with a Creative Commons CC-BY license. Endoscopy E-Videos qualify for HINARI discounts and waivers and eligibility is automatically checked during the submission process. We grant 100% waivers to articles whose corresponding authors are based in Group A countries and 50% waivers to those who are based in Group B countries as classified by Research4Life (see: https://www.research4life.org/access/eligibility/ ). This section has its own submission website at https://mc.manuscriptcentral.com/e-videos .
